# 153. Molnupiravir Exhibits a High Barrier to the Development of SARS-CoV-2 Resistance in vitro

**DOI:** 10.1093/ofid/ofac492.231

**Published:** 2022-12-15

**Authors:** Julie Strizki, Nicholas Murgolo, John Gaspar, John Howe, Beth Hutchins, Hiroshi Mohri, David D Ho, Daria Hazuda, Jay Grobler

**Affiliations:** Merck & Co., Inc., West Point, Pennsylvania; Merck & Co., Inc., West Point, Pennsylvania; Merck and Co., Boston, Massachusetts; Merck and Co., Boston, Massachusetts; Merck and Co,, Rahway, New Jersey; Aaron Diamond Research Institute, Columbia University, New York, New York; Columbia University Vagelos College of Physicians and Surgeons, New York, NY; Merck & Co., Inc., West Point, Pennsylvania; Merck & Co., Inc., West Point, Pennsylvania

## Abstract

**Background:**

Molnupiravir is an orally available prodrug of the antiviral nucleoside analog N-Hydroxycytidine (NHC). In preclinical studies NHC has shown broad-spectrum antiviral activity against multiple RNA viruses including SARS-CoV-2. Incorporation of NHC by viral polymerases impairs replication by introducing errors into the viral genome. NHC has been shown to have a high barrier to the development of resistance in vitro with RSV, Influenza and Venezualen Equine Encephalitis viruses. In these studies, we have explored the potential for SARS-CoV-2 to develop resistance to NHC in cell culture.

**Methods:**

Vero E6 cells were infected with SARS-CoV-2 (WA-1) in triplicate in the presence of NHC or a C3L-protease inhibitor (MRK-A). Culture supernatants from wells with the highest drug concentration exhibiting a cytopathic effect (CPE) score of ≥ 2+ were repassaged and at each passage, IC50 values were estimated based on CPE scoring. At each passage, full genome next generation sequencing (NGS) was performed on the viral RNA

**Results:**

No change in susceptibility to NHC (EC50 fold change ≤ 1.1) was noted in 2 of 3 cultures and a 2-fold change was observed in one culture after 30 passages. In contrast, a 3- to 4-fold decreases in susceptibility to the 3CL protease inhibitor were seen by passage by 12, with increasing resistance of 4.6- to 15.7-fold observed by passage 30. NHC passaged viruses exhibited 53 to 99 amino acid changes, including substitutions and deletions (both in-frame and frameshift), across 25 different viral proteins as compared with 10 to 13 changes in 13 proteins in the MRK-A cultures. With NHC, 3 to 4 changes were observed in the viral polymerase; however, these were randomly distributed, and none were observed more than once. In contrast, the 3CL protease passaged virus had a nsp5 T21I substitution detected in all 3 cultures.

**Conclusion:**

No evidence of SARS-CoV-2 phenotypic or genotypic resistance was observed following 30 passages with NHC. A random pattern of amino acid changes were observed across multiple proteins consistent with the mechanism of action of NHC. In the same study, resistance was readily selected to a control 3CL protease inhibitor. Together these data support previous reports demonstrating the high barrier to resistance of NHC.

**Disclosures:**

**Julie Strizki, PhD**, Merck and Co.: Stocks/Bonds **Nicholas Murgolo, PhD**, MERCK: Employee|MERCK: Stocks/Bonds **John Howe, PhD**, Merck & Co Inc: Employee|Merck & Co Inc: Stocks/Bonds **Beth Hutchins, PhD**, Merck & Co.: Employee|Merck & Co.: Stocks/Bonds **Hiroshi Mohri, PhD/MD**, Merck: Grant/Research Support **Daria Hazuda, PhD**, Merck: Employee|Merck: Stocks/Bonds **Jay Grobler, PhD**, Merck & Co., Inc.: Employee|Merck & Co., Inc.: Stocks/Bonds.

